# Effects of Allopolyploidization and Homoeologous Chromosomal Segment Exchange on Homoeolog Expression in a Synthetic Allotetraploid Wheat under Variable Environmental Conditions

**DOI:** 10.3390/plants12173111

**Published:** 2023-08-30

**Authors:** Zhibin Zhang, Ruili Lv, Bin Wang, Hongwei Xun, Bao Liu, Chunming Xu

**Affiliations:** 1 Key Laboratory of Molecular Epigenetics of the Ministry of Education (MOE), Northeast Normal University, Changchun 130024, China; 2 Northeast Institute of Geography and Agroecology, Chinese Academy of Sciences (CAS), Changchun 130102, China

**Keywords:** allopolyploidy, genome shock, subgenome expression, homoeologous chromosomal segment exchange, genome evolution, nascent plant allopolyploids

## Abstract

Allopolyploidy through the combination of divergent genomes into a common nucleus at doubled dosage is known as a potent genetic and evolutionary force. As a macromutation, a striking feature of allopolyploidy in comparison with other mutational processes is that ‘genome shock’ can be evoked, thereby generating rapid and saltational biological consequences. A major manifestation of genome shock is genome-wide gene expression rewiring, which previously remained to be fully elucidated. Here, using a large set of RNAseq-based transcriptomic data of a synthetic allotetraploid wheat (genome AADD) and its parental species, we performed in-depth analyses of changes in the genome-wide gene expression under diverse environmental conditions at the subgenome (homoeolog) level and investigated the additional effects of homoeologous chromosomal segment exchanges (abbreviated HEs). We show that allopolyploidy caused large-scale changes in gene expression that were variable across the conditions and exacerbated by both stresses and HEs. Moreover, although both subgenomes (A and D) showed clear commonality in the changes, they responded differentially under variable conditions. The subgenome- and condition-dependent differentially expressed genes were enriched for different gene ontology terms implicating different biological functions. Our results provide new insights into the direct impacts of allopolyploidy on condition-dependent changes in subgenome expression and the additional effects of HEs in nascent allopolyploidy.

## 1. Introduction

Polyploidization, i.e., whole-genome duplication (WGD), is a prominent intrinsic force driving the speciation and evolution of many biological taxa on Earth. In the course of plant evolutionary history, all angiosperms have undergone at least two rounds of WGD [1]. The first WGD event took place among the common ancestors of seed plants approximately 319 million years ago (MYA), while the second event occurred among the common ancestor of angiosperms ca. 192 MYA [1]. Extensive research has established that multiple extant plant lineages experienced additional WGDs, many of which coincided with the mass extinction period known as the Cretaceous–Tertiary (KT) boundary [2]. This intriguing ‘coincidence’ suggests that polyploidization may have played a pivotal role in the survival and adaptation of plants during catastrophic environmental conditions [2].

Notably, plant WGD is often accompanied by hybridization, a process known as allopolyploidization [3]. Allopolyploids, by integrating and doubling two genetically and epigenetically diverged genomes, often lead to ‘genomic shock’ that may result in profound changes in karyotype, DNA sequence, chromatin modification, gene expression, and phenotype [3,4,5,6,7,8,9,10,11,12,13,14]. A large body of studies in several plant species has reported numerous non-additively expressed genes that deviate from the expected mid-parental values in artificially synthesized or newly formed natural allopolyploids, such as those in Arabidopsis, wheat, and coffee. These genes exhibit diverse novel expression patterns, including parental expression level dominance, subgenome expression dominance, and transgressive expression [15]. On the genome-wide scale, subgenome expression dominance appears to be a common feature in newly synthesized or recently formed natural plant allopolyploids, although exceptions have also been documented [11,15,16].

Artificially synthesized allopolyploids have been widely employed in research aimed at understanding the immediate impacts of allopolyploidization on gene expression, due to their well-defined parental genome information and the absence of confounding effects of other evolutionary forces, e.g., selection and drift. Furthermore, with the rapid advancement in analytical methods, transcriptome sequencing has been utilized to investigate the expression of homoeologs in newly synthesized allopolyploids. The presence of homoeolog expression bias has been observed in diverse synthetic and recently formed natural allopolyploids [17,18]. While a significant portion of homoeolog expression biases in newly synthesized polyploids can be attributed to inherited expression differences from their diploid parents (i.e., parental legacy), there are also numerous instances of novel homoeolog expression patterns arising from transcriptional regulatory interactions between subgenomes [17,18]. Furthermore, it has been reported that the expression level dominance in cotton allopolyploids is primarily associated with the up- or down-regulation of the homoeolog from the non-dominant parent [17]. The extent of homoeolog expression bias in allopolyploids was found to increase with evolutionary time [17,18,19]. Importantly, the genomic shock-incurred rapid changes in gene expression may have significant longer-term implications in growth, development, and adaptation, thus potentiating a broader range of phenotypic flexibility and adaptability relative to their diploid progenitors.

A study in cotton demonstrated that relative homoeologous expression underwent changes during organ development and in response to stresses [20]. Another investigation in cotton revealed major effects of abiotic stresses on a set of preselected duplicate gene expressions in allopolyploids, with the magnitude of these effects varying, depending on the specific genes, stress types, and organs [21]. In contrast, studies in coffee showed that, while the genomic expression level dominance was strongly influenced by growth temperatures [22], the relative homoeologous expression exhibited only moderate variations across organs and different growth conditions [23,24]. A recent study in cotton confirmed that allopolyploids indeed possess a wide-ranging stress response flexibility compared to their diploid counterparts, most likely being mediated by complex suites of duplicated genes and regulatory factors [25]. Currently, limited research has been conducted on the effects of various stress conditions on homoeolog expression resulting from allopolyploidization. Therefore, it is necessary to conduct further comparative studies on homoeolog expression in newly synthesized allopolyploids, in comparison with their direct parents, under different stress conditions in various plant species, to elucidate the possible common trends of gene expression changes resulting from plant allopolyploidization.

Common wheat (*Triticum aestivum*) is a complex polyploid species consisting of three distinct subgenomes: A, derived from *T. urartu* in the A lineage; B, derived from an unknown species closely related to the extant *Aegilops speltoides* in the B lineage; and D, derived from *Ae. tauschii* in the D lineage. This intricate composition, including the combination of three subgenomes, was formed through two consecutive rounds of allopolyploidization events [26,27,28]. In our previous studies, we successfully produced allotetraploid wheat (AADD and DDAA) by crossing the still-existing direct diploid progenitor species, *T. urartu* (AA) and *Ae. tauschii* (DD), which were the diploid progenitors of common wheat (*T. aestivum*, BBAADD). We found that during the inflorescence development of the allotetraploids (AADD or DDAA), the two subgenomes (AA and DD) expressed differentially with relative subgenome expression dominance mirroring the inflorescence and spike morphologies [29]. Furthermore, from a specific selfed lineage of AADD, which we termed AT3-EUP in our prior study [30] (renamed as EUP in this investigation for clarity), we identified a specific line named AT3-F2-HOT-1-Line (renamed as X190 in this study for clarity) that exhibited copy number variations due to homoeologous chromosomal segment exchanges (HEs) and which manifested significantly enhanced tolerance to salinity and hyper-osmotic (mimic drought) stresses relative to its isogenic euploid, AT3-EUP [30]. Transcriptomic analysis of X190 revealed an up-regulation of stress-responsive genes within the HE regions, suggesting a potential causation of the higher expression of these copy-number-varied focal genes to the improved abiotic stress tolerance in X190 [30]. 

Building on the aforementioned prior findings, the present study aimed to take advantage of the large set of transcriptomic data generated from our previous study [30] to perform a comprehensive investigation of subgenome (homoeolog)-specific expression changes in the newly synthesized allotetraploid wheat lines with and without HEs in comparison with its diploid parental species under various environmental conditions. We investigated the numbers, proportions, and functional enrichment of differentially expressed genes (DEGs) in the two subgenomes of the allotetraploid wheat lines, relative to their parental genomes (represented by a mixture of equal amounts of parental RNAseq data), and analyzed the regulatory mechanisms underlying the homoeolog expression patterns following allopolyploidization and HEs, thereby elucidating the interplays between relative homoeolog expression dynamics in response to environmental conditions in allopolyploids and the additional impacts of secondary structural changes (i.e., HEs) that occurred in the allotetraploids. Our results provide new insights into the direct impacts of allopolyploidy on environmental condition-dependent changes in subgenome expression and unravel significant additional effects of HEs superimposed on the impacts’ nascent allopolyploidization. 

## 2. Results

### 2.1. Subgenome Expression Changes following Allopolyploidization with and without Homoeologous Chromosomal Segment Exchanges under Different Environmental Conditions

**Figure 1:**
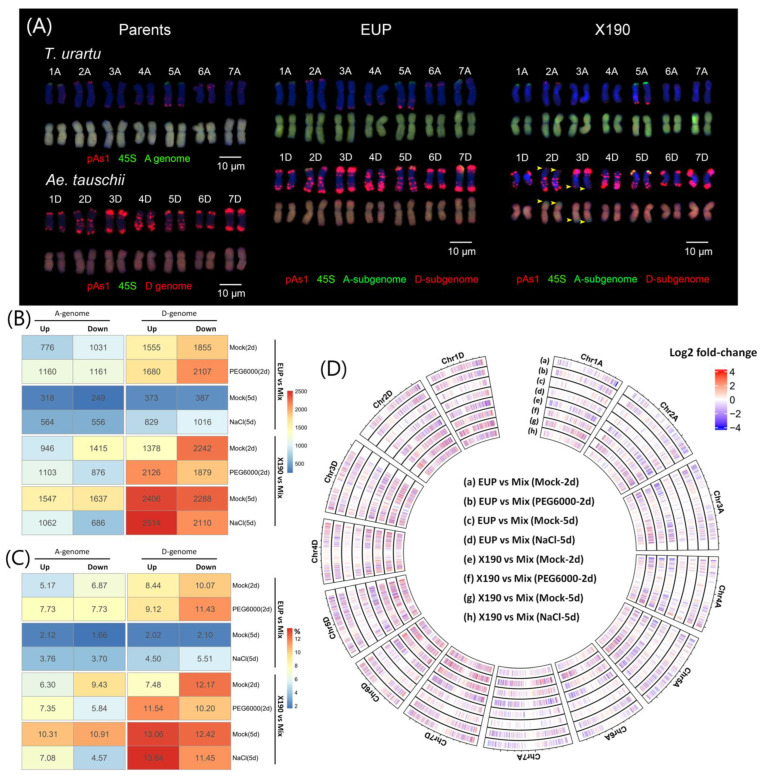
Karyotypes of the diploid parents and the two synthetic allotetraploid wheat lines, a euploid line (EUP) and a homoeologous exchange (HE)-containing line (X190), and summary of differentially expressed genes (DEGs) between each of the two allotetraploid lines and their parents (Mix) under varied conditions. (**A**) GISH/FISH-based karyotypes of parents and the synthetic tetraploid wheat. The two HEs in X190 are indicated by arrowheads. (**B**) Numbers of up- and down-regulated DEGs in A and D subgenomes. (**C**) Proportions of up- and down-regulated DEGs in A and D subgenomes. (**D**) Chromosomal distribution of DEGs, wherein each vertical line represents a DEG, and its color represents the log2 fold-change of expression ratio compared to Mix.

To address the impacts of allopolyploidization per se and its attendant secondary structural chromosomal variation on gene expression, we first re-verified the identities of the two synthetic allotetraploid wheat lines (AADD), a euploid and a homoeologous exchange (HEs)-containing line, through the combined use of fluorescence in situ hybridization (FISH) and genomic in situ hybridization (GISH), as detailed in [30]. Compared to the euploid (EUP) allotetraploid line, the HE-containing line (X190) involved two homozygous HE events: the first event featured the replacement of the end region of the short arm of chromosome 2D (2DL) with the end region of the short arm of chromosome 2A (2AS) (designated as 2DS-ter(0)/2AS-ter(4)), and the second event involved the replacement of the end region of 3DL with the end region of 3AL (designated as 3DL-ter(0)/3AL-ter(4)) (Figure 1A). We compared the gene expression levels between each of the two allotetraploid wheat lines and their parents (Mix, represented by a mixture of equal amounts of AA and DD RNAseq data) under four conditions: mock hyper-osmotic treatment of 2 days (designated as mock-2d), hyper-osmotic treatment of 2 days (designated as PEG6000-2d), mock salinity treatment of 5 days (designated as mock-5d), and salinity treatment of 5 days (designated as NaCl-5d). The analyses were focused on identifying the numbers and proportions of differentially expressed genes (DEGs) and examining their chromosomal distribution within the A and D subgenomes under the variable conditions. The results revealed that the numbers of DEGs between the euploid (EUP) and the parents (Mix) varied widely, ranging from 1327 to 6108 across the four conditions (Figure 1B, Table S1). Notably, the numbers of DEGs between the HE-containing offspring (X190) and the parents (Mix) were further exacerbated, ranging from 5981 to 7878 (Figure 1B, Table S1). 

Interestingly, the numbers of DEGs between EUP and Mix were higher under both stress conditions (6108 under drought-2d and 2965 under salt-5d) compared to their corresponding mock conditions (5217 under mock-2d and 1327 under mock-5d) (Figure 1B). Surprisingly, X190 exhibited the opposite trend, with a lower number of DEGs under salt-5d condition (6372) compared to the control conditions (7878 under mock-5d) (Figure 1B, Table S1) and nearly the same numbers of DEGs under drought-2d (5984) and mock-2d (5981). These results suggest that environmental variables had a marked impact on the differential expression of genes between the newly synthesized allotetraploid wheat and their parents, and distinct patterns or even opposing trends may emerge in the HE-containing progenies.

Comparing the numbers and proportions of DEGs between the A and D subgenomes showed that, across all conditions, the numbers and proportions of DEGs in both allotetraploid lines (EUP and X190) relative to the parental Mix were consistently higher in the D than in the A subgenomes (Figure 1B,C). This indicates that allopolyploidization had a greater impact on gene expression in the D subgenome than in the A subgenome. Moreover, the differences in the proportions of up- and down-regulated DEGs in the D subgenome of X190 were generally higher than those in EUP under the corresponding conditions (Figure 1C, Table S2). In addition, the analysis of regulatory types (up- and downregulation) of DEGs revealed a consistent pattern, that is, under all conditions, the EUP exhibited more down-regulated DEGs (ranging from 2.10% to 11.43%) than up-regulated DEGs (ranging from 2.02% to 9.12%) in the D subgenome (Figure 1C). There was no consistent pattern in the numbers and proportions of up- and down-regulated DEGs in the A subgenome in EUP vs. Mix across the conditions (Figure 1B,C). In contrast to EUP, there were more up- than down-regulated DEGs in the D subgenome in X190 under all conditions except for the mock-2d condition (Figure 1B,C). This observation indicates a potential role of HEs in modulating and generating new homoeolog expression patterns in allopolyploids, which might contribute to its higher tolerance to the stresses in addition to the focal genes within the HEs [30]. 

We next examined the chromosomal distribution of the subgenome DEGs. We found that across most treatment conditions, the counts of up- and down-regulated DEGs in the A and D subgenomes exhibited a relatively uniform distribution along their respective chromosomes (Figure 1D; Figure S1). However, in X190, there were specific conditions under which individual chromosomes showed a higher number of DEGs. For instance, under the mock-2d condition, there was a significantly higher count of down-regulated DEGs (404) than up-regulated DEGs (181) on Chr5D. Similarly, under the NaCl-5d condition, there was a significantly higher count of up-regulated DEGs (448) than down-regulated DEGs (329) on Chr5D (Figure 1D; Figure S1).

### 2.2. Functional Enrichment of Subgenome DEGs in the Synthetic Allotetraploid Wheat

To explore the possible functional relevance of allopolyploidization- and HE-induced subgenome differential expression in response to the diverse environmental conditions, we conducted Gene Ontology (GO) enrichment analyses of the sets of DEGs between each of the two synthetic allotetraploid wheat lines and their diploid parents in the A genome (parent)/subgenome (allotetraploid) and D genome (parent)/D subgenome (allotetraploid), in separation. The analyses revealed significant enrichment of multiple common, as well as distinct, GO terms by the different sets of DEGs.

**Figure 2:**
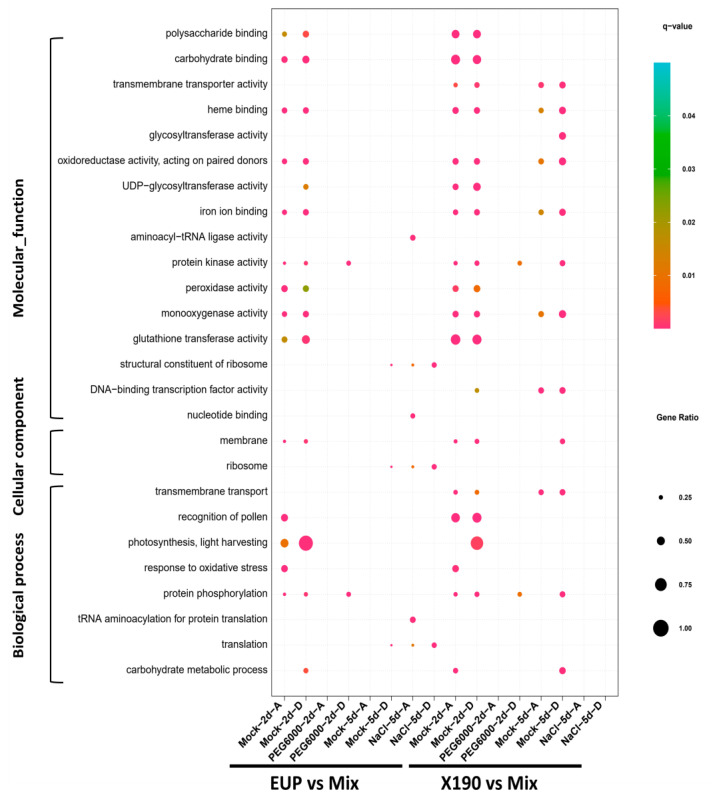
Over-represented gene ontology (GO) terms in DEGs between the synthetic allotetraploid wheat and its parents under different conditions. The 10 most-enriched GO terms are shown if the over-represented terms exceed 10.

First, in EUP vs. Mix under the mock-2d condition, we identified 26 significantly over-represented GO terms in the DEGs of the A subgenome vs. A genome, while 23 GO terms were significantly over-represented in the DEGs of the D subgenome vs. D genome (Supplementary Dataset). Notably, a considerable number of GO terms showed concurrent enrichment in both sets of the A and D subgenome vs. A and D genome DEGs, indicating a common impact on both subgenomes of allopolyploidization on the expression of genes associated with these specific biological processes and molecular functions (Figure 2). Nonetheless, certain GO terms, such as response to oxidative stress, were significantly over-represented solely in the DEGs of the A subgenome vs. A genome (Figure 2). Conversely, fewer GO terms exhibited significant enrichment in the DEGs of EUP vs. Mix under other conditions, suggesting the existence of condition-dependent differences in the functional enrichment of DEGs in EUP vs. Mix. Furthermore, in X190 vs. Mix, the over-represented GO terms and their distribution patterns among different conditions closely resembled those observed in EUP vs. Mix for both the A and D subgenomes vs. genomes, except for a higher prevalence of significantly over-represented GO terms in the DEGs under the mock-5d condition (Figure 2).

We conducted further analysis to investigate the impact of environmental stresses on gene expression at the subgenome level and observed extensive expression repatterning in both EUP and X190 under the stress conditions (Figure 2). In the EUP, compared to the normal condition, only the Gene Ontology (GO) terms related to protein phosphorylation and protein kinase activity (DEGs in the D subgenome) showed enrichment in hyper-osmotic conditions. In the salinity treatment, the GO terms associated with translation, ribosome, and nucleotide binding (DEGs in the D subgenome) exhibited enrichment. Similar to EUP, X190 exhibited a comparable pattern, wherein only GO terms involved in protein phosphorylation and protein kinase activity (DEGs in the D subgenome) maintained enrichment under hyper-osmotic conditions. Notably, no enriched GO terms were observed in any of the stress conditions for both EUP and X190. 

### 2.3. Modes of Homoeolog Expression in the Synthetic Allotetraploid Wheat

To further investigate the impact of allopolyploidization and its attendant HEs on homoeologous expression patterns, we identified the 1:1 homoeologous gene pairs between the A and D subgenomes and conducted a comparative expression analysis between copies of the homoeologous gene pairs (see Section 4). The homoeologous gene pairs were categorized into nine modes belonging to four regulation types, based on changes in the relative homoeolog expression in the allotetraploids in relation to the genome expression in the parents. The results showed that, across all conditions, the majority of homoeologous gene pairs (>70%) exhibited conserved regulation (modes 1 to 3) in both EUP and X190, indicating that a significant portion of parental expression differences were stably transmitted to and maintained in the synthetic allotetraploid wheat following allopolyploidization (Table 1). Additionally, the proportions of homoeologous gene pairs showing convergent regulation (12.15–18.98%) were higher than those showing divergent regulation (7.62–13.24%) under all the conditions, suggesting that allopolyploidization exerts a stronger common trans-regulation on both homoeologous gene pairs compared to cis-regulation on subgenome-specific expression [31] upon the merging and doubling of the two parental genomes (Table 1). The proportion of homoeologous gene pairs with reversed regulation was consistently the lowest, accounting for less than 0.5% of the analyzed homoeologous gene pairs (Table 1). Notably, in all these aspects, the HE-containing and HE-free allotetraploids did not show qualitative differences that otherwise should be readily discernible.

By comparing the stress and mock control conditions, we observed that the proportions of homoeologous gene pairs in modes 1 and 2 (parents A > D/progeny A > D, parents A < D/progeny A < D) were higher under the two stress conditions (PEG6000-2 and NaCl-5d) than under the respective control conditions (mock-2d and mock-5d) in both allotetraploid lines (Table 1). This indicates that the stress conditions increased the expression differences between the homoeologous gene pairs, and the regulatory mechanisms in response to the stresses were inherited by the synthetic allotetraploid wheat.

Furthermore, in EUP, under the two stress conditions (PEG6000-2d and NaCl-5d), the numbers of homoeologous gene pairs exhibiting convergent regulation (modes 4 and 5) and divergent regulation (modes 6 and 7) were higher than those under their respective mock control conditions (mock-2d and mock-5d) (Table 1). This suggests that the stress conditions augmented the proportions of the expression of parental non-conserved homoeologous gene pairs in the allotetraploids relative to those between the parents. Again, in this aspect, the HE-containing and HE-free allotetraploid lines did not differ qualitatively (Table 1).

### 2.4. Homoeolog Expression Regulation in the Synthetic Allotetraploid Wheat

**Figure 3:**
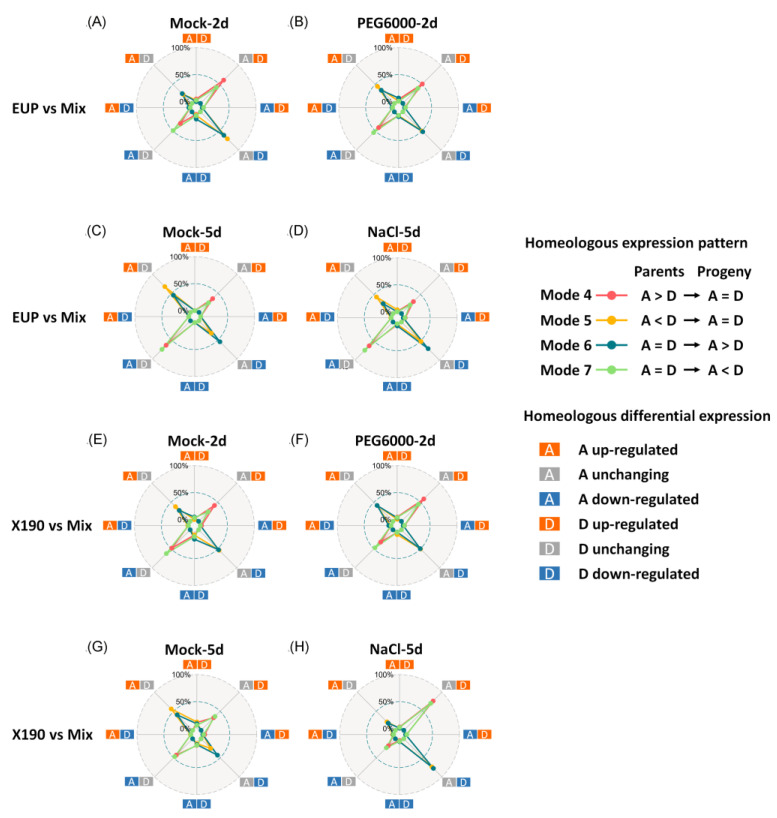
Relationships between relative homoeologous expression modes and homoeologous expression changes.

To gain further insights into the mechanisms underlying convergent and divergent regulation in the synthetic allotetraploid wheat, we conducted an in-depth analysis of expression changes in homoeologous gene pairs between the allotetraploid wheat lines and their parental mix. We found that, in EUP, the majority of homoeologous gene pairs exhibiting convergent regulation (modes 4 and 5) or divergent regulation (modes 6 and 7) were associated with expression changes in only one copy of a given homoeologous gene pair, while the other copy remained unchanged (Figure 3, Table S3). Notably, the relative contributions of the A and D subgenome DEGs to changes in homoeolog expression modes varied under different conditions. For instance, in mode 4 under the mock-2d condition, more D than A subgenome DEGs were involved, whereas, in mode 4 under the NaCl-5d condition, more A than D subgenome DEGs contributed to the homoeologous relative expression changes. Moreover, we observed that, under certain conditions and homoeologous expression modes, a small number of homoeologous gene pairs exhibited simultaneous up- or down-regulation in both copies in the allotetraploids. However, homoeologous gene pairs displaying opposite regulation in both copies were rare. The relationship between homoeolog expression modes and DEGs in both the HE-free EUP and the HE-containing X190 were largely similar. Notwithstanding, we noted that under the NaCl-5d condition, while expression changes in the D subgenome copies accounted for nearly all the homoeologous expression modes in X190, this was not the case in EUP (Figure 3, Table S4). 

## 3. Discussion

Allopolyploidy, through the merging and doubling of related but diverged genomes of different species, may instigate an array of rapid changes collectively termed ‘genome shock’, which includes, but is not limited to, alterations in gene expression, chromosome rearrangement, and epigenetics chromatin modification [3,4,5,6,7,8,9,10,11,13]. Among the changes, alteration in gene expression appears to be the most common manifestation [10,15,16,17]. Alteration of gene expression is reflected in various forms, including nonadditivity, transgressivity, overall expression level dominance, and subgenome expression dominance, etc. [10,32]. It has also been documented or suggested that genome shock-induced gene expression alterations are not only important in mitigating subgenome incompatibilities but also play roles in the longer-term adaptation of allopolyploids to more diverse environmental conditions than their diploid progenitors [33,34]. Nevertheless, the biological meanings of genome shock-induced gene expression changes remain to be fully understood, in part because, in naturally formed allopolyploid species, it is difficult to deconvolute the shock-induced expression alterations from those accumulated due to post-allopolyploidy evolutionary changes. It is also difficult to distinguish de novo altered expression patterns from those inherited from parents (parental legacy), as the exact parents may have gone extinct or, at least, are difficult to determine. Furthermore, even less is understood about the immediate impacts of genome shock-induced secondary structural chromosomal changes on gene expression, which are superimposed on the effects of allopolyploidization, although they are known to occur frequently in newly formed allopolyploids due to meiosis instability [34,35,36]. This again is difficult to study using naturally formed allopolyploid species, as structural variants are known to affect recombination and hence mutational trajectories [37,38]. Because of these limitations, synthetic allopolyploids are suitable systems to address the above-raised two questions. Indeed, using synthetic allopolyploids in diverse plant taxa, it was found that genome shock-induced gene expression changes play important roles in phenotypic novelty and adaptation to stressful conditions [39,40,41]. In contrast, only in circumstantial instances have the direct additional effects of homoeologous chromosomal segment exchanges (HEs) been studied [30,42,43]. In both aspects, how gene expression changes on the genome-wide scale at the subgenome (homoeologue) level, and its relation to response to different environmental conditions, are poorly understood. 

In this study, we took advantage of a large set of RNAseq-based transcriptomic data that we generated previously in two synthetic allotetraploid wheat lines (genome AADD), which contained or did not contain homoeologous chromosomal segment exchanges (HEs), respectively, together with their exact diploid parents [30]. Using these data, we addressed both the effects of allopolyploidization per se and the additional effects of HEs superimposed on those of allopolyploidization on subgenome (homoeologue) expression in response to diverse environmental conditions. For differentially expressed genes (DEGs), we find that, in comparison with the diploid parents, allotetraploidization per se induced extensive changes in gene expression on the genome-wide scale and at the subgenome level, while the HEs generated additional changes. These observations are consistent with previous findings [42,43]. However, we find that the numbers, proportions, and functional enrichment of differentially expressed genes (DEGs) in the two subgenomes of the allotetraploid wheat lines relative to their parental genomes are more different than similar, regardless of the presence or absence of the HEs (Figure 1 and Figure 2). Moreover, different chromosomes also exhibited distinct responses responded differently with respect to the effects of allopolyploidization and its attendant HEs under different conditions. For example, chromosome 5D showed markedly more DEGs than the other chromosomes in the HE-containing line under several conditions (Figure 1D; Figure S1). These results have extended our recent findings that, in the synthetic allotetraploid wheat with AADD or DDAA genomes, differential subgenome expression dynamics in the course of growth and reproductive development under normal conditions mirrors their spike morphology and is, at least in part, related to the manifestation of biomass and seed production heterosis seen in the allotetraploids [29]. In addition, the substantial differences in subgenome vs. parental genome comparisons between the two allotetraploid lines with and without HEs may suggest that, apart from the focal genes mapped within the HE regions whose copy numbers are altered, the large set of DEGs that are mapped genome-wide without copy number alterations is likely to contribute to the enhanced stress tolerance in the HE-containing line [30]. 

At the level of subgenome, i.e., homoeologous gene pairs, previous studies indicate that biotic and abiotic stresses play a crucial role in shaping their expression pattern response to allopolyploidization [17,21,44]. Consistent with these prior findings, our study revealed significant variations in expression patterns among homoeologous gene pairs under different conditions, although the HE-containing and HE-free allotetraploids did not show significant differences (Table 1). While the highest proportion of homoeologous gene pairs exhibited conserved regulation, it is noteworthy that, under stress conditions, there was a tendency for homoeologous gene pairs to lose their conserved regulation. This resulted in an increased occurrence of homoeologous gene pairs with non-conserved regulation, including both convergent and divergent regulations. The influence of stress conditions on the regulatory dynamics of homoeologous gene pairs highlights the potential for altered expression patterns and regulatory mechanisms in response to environmental challenges [25,45,46]. Notably, the dramatic changes observed in expression patterns are primarily attributed to alterations in only one homoeologous copy of a given subgenome, rather than changes occurring in both copies simultaneously (Figure 3). This divergence in response and regulation between the two subgenomes may be influenced by their asymmetrical epigenetic modifications. These epigenetic modifications are likely to contribute to the distinct regulatory landscapes of the subgenomes, resulting in the observed differences in expression patterns between the homoeologs [47,48]. Further understanding the role of epigenetic modifications in shaping the regulatory dynamics of homoeologous gene pairs may provide insights into the complex regulatory mechanisms underlying allopolyploidization in wheat and other allopolyploid crops. 

Altogether, our results provide new insights into the direct impacts of allopolyploidy on environmental condition-dependent changes in subgenome expression and unravel significant additional effects of HEs overlaid on the impacts of nascent allopolyploidization. Further studies are needed to pinpoint some large-effect DEGs and establish their causality to adaptation to various environments by allopolyploidy. 

## 4. Materials and Methods

### 4.1. Plant Materials 

A euploid synthetic allotetraploid wheat line with AADD genomes was generated through interspecific hybridization between *Triticum urartu* (AA) and *Aegilops tauschii* (DD), followed by the induction of chromosome doubling in the F1 hybrids using colchicine [30]. In the course of propagation, we identified a line in which homoeologous chromosomal segment exchanges (HEs) occurred, and which was homozygotized to become a homogeneous HE-containing line [30]. For this study, we verified both allotetraploid lines and their parents by combined use of fluorescence in situ hybridization (FISH) and genomic in situ hybridization (GISH). For the FISH analysis, the 45S ribosomal DNA (rDNA) and the tandem repeat pAs1 [49] were labeled with Alexa Fluor 488-5-dUTP (green) and Texas red-5-dCTP (red), respectively. This labeling allowed the identification of each of the 14 chromosome pairs of AT3. For the GISH analysis, genomic DNA from T. urartu (AA) and Ae. tauschii (DD) were separately labeled with either Alexa Fluor 488-5-dUTP (green) or Texas red-5-dCTP (red). These labels were used to differentiate between the two subgenomes. 

### 4.2. Data Processing and Gene Expression Counts

The RNAseq data used in this study were generated previously [30], which involved the data of a euploid synthetic allotetraploid wheat line designated as EUP (AT3-Eup in [30]), an HE-containing line designated as X190 (AT3-F2-HOT-1-Lines in [30]), and their diploid parents (Figure 1D). Each genotype was subjected to two sets of mock and treatment conditions involving treatments by 200 mM NaCl (for 5 days) and 20% PEG6000 (for 2 days), which served as salinity and hyper-osmotic stresses, respectively [30]. 

The RNAseq data were trimmed using Trimmomatic (version 0.39) per the following parameters: “LEADING:5 TRAILING:5 HEADCROP:10 MINLEN:75 TOPHRED33”. Then, the clean data were mapped to a ‘concatenated reference genome’ that was created by merging the reference genomes of the two diploid parental species *Triticum urartu* (AA) and *Aegilops tauschii* (DD) using HISAT2 (version 2.1.0) [50]. The resulting concatenated reference genome comprised a total of 76,350 genes, with 37,568 genes from the AA reference genome and 38,782 genes from the DD reference genome. The raw read counts for the genes were calculated using the feature Count (version 2.0.4) for each sample. The genes falling in known HE regions in X190 (Chr2A: 0.8–51.8 Mb, 1074 genes; Chr2D: 0.4–58.0 Mb, 1075 genes; Chr3A: 684.6 Mb-end, 1188 genes; Chr3D: 557.4 Mb-end, 1250 genes) or in scaffolds without chromosome information were excluded from further analyses. We removed the homoeologous genes in the HE regions due to their duplicated or deleted copy numbers. This step was crucial to prevent an imbalanced number of differentially expressed genes (DEGs) in the HE regions, which could affect the comparison of DEGs between different conditions (normal, hyper-osmotic, and salinity conditions).

### 4.3. Identification of Differentially Expressed Genes

Differential expression analysis was performed to identify genes showing significant expression differences between each of the two allotetraploid lines that were HE-free (EUP) or containing HEs (X190) and their diploid parents (Mix). Genes with an average read count below 10 or above 5000 across all the samples were filtered out. This filtering step resulted in a final set of 15,015 *T. urartu* genes and 18,438 *Ae. tauschii* genes that were retained for subsequent analyses. DESeq2 was employed to analyze the gene expression differences between the allotetraploids and parents under each condition [51]. The raw *p*-values were adjusted using the Benjamini–Hochberg (BH) method [52], and the genes with adjusted *p*-values < 0.05 were considered as differentially expressed genes (DEGs).

### 4.4. Gene Ontology Enrichment Analysis

Gene Ontology (GO) enrichment analysis was conducted to explore the possible functional relevance of the DEGs. The protein sequences of all the genes of *T. urartu* and *Ae. tauschii* were annotated using InterproScan (version 5.52) with the default settings to obtain their GO annotations. The subsequent GO enrichment analysis was performed separately for the DEGs identified in the A and D subgenomes. To ensure robust results, GO terms that had fewer than five expressed genes were excluded from further analysis. To assess whether a particular GO term was over-represented among the DEGs, a one-tailed hypergeometric test was employed. This statistical test evaluated the likelihood of observing an enrichment of a specific GO term within the DEG set. The raw p-values obtained were subsequently adjusted for multiple testing using the Benjamini–Hochberg (BH) method [52]. Only those GO terms with adjusted *p*-values less than 0.05 were considered significantly over-represented.

### 4.5. Identification of Collinear Homoeologous Gene Pairs 

The genome-wide orthologues between *T. urartu* and *Ae. tauschii* were identified using OrthoFinder (version 2.5.5) [53] with the default parameters. The collinear blocks between the *T. urartu* and *Ae. tauschii* genomes were identified using MCScanX [54] with default parameters. The one-to-one orthologous gene pairs showing collinearity between *T. urartu* and *Ae. tauschii* were selected and defined as homoeologous gene pairs for further analysis.

### 4.6. Comparison of Homoeologous Expression and Classification of Homoeologous Expression Modes

To maintain the homoeologous gene pairs for further analysis, we filtered out pairs with transcript length differences exceeding 10% to mitigate the influence of length disparity. This resulted in a total of 6017 retained homoeologous gene pairs. For the analysis of homoeologous expression patterns, we focused on pairs that were expressed in at least one subgenome. By applying this additional criterion, we narrowed down our selection to 4420 homoeologous gene pairs that met both the transcript length and expression threshold requirements. These refined pairs were used for the subsequent analysis of homoeologous expression patterns. DESeq2 was utilized to analyze the expression differences between the copies of A and D genomes within the homoeologous gene pairs, as well as the expression differences between the diploid parents and tetraploid progeny for copies from either genome.

By comparing the expression differences between homoeologous copies in parents and progeny under the same condition, the homoeologous gene pairs were classified into nine distinct patterns. In mode 1 to mode 3, the A and D copies of homoeologous gene pairs exhibited the same differential expression pattern before and after allopolyploidization. In mode 4 and mode 5, the A and D copies showed significant expression differences between two diploid parents, but these differences became insignificant after allopolyploidization. In mode 6 and mode 7, the A and D genome copies did not exhibit significant expression differences between parents, but significant expression differences emerged after allopolyploidization. In mode 8 and mode 9, the A and D genome copies displayed opposite differential expression patterns between parents and exhibited contrasting expression differences during allopolyploidization.

## 5. Conclusions

In summary, this study illuminates the intricate interplay among allopolyploidization, HEs, environmental conditions, and homoeologous expression in a synthetic allotetraploid wheat. Our findings reveal that allopolyploidy induces substantial alterations in gene expression, which vary across environmental conditions. The two subgenomes do not behave the same in response to the stress conditions, with the D subgenome being more responsive than the A subgenome. Moreover, our results demonstrate the stability of parental expression differences in most homoeologous gene pairs, which are faithfully transmitted and maintained in the synthetic allotetraploid wheat. Additionally, allopolyploidization exerts a stronger trans-regulatory effect than cis-regulation on the expression of homoeologous gene pairs. Furthermore, diverse environmental conditions impact the expression modes of homoeologous gene pairs, with the majority of homoeologous gene pairs exhibiting convergent or divergent regulation associated with expression changes in only one copy of a given homoeologous gene pair. Our study provides new insights into the direct consequences of allopolyploidy on gene expression and the additional effects of HEs under variable environmental conditions.

## Figures and Tables

**Table 1:** Summary of homoeologous expression modes in the synthetic allotetraploid wheat.

Mode		Progeny	Mix vs. EUP				Mix vs. X190			
	Parents		Mock 2d	PEG6000 2d	Mock 5d	NaCl 5d	Mock 2d	PEG6000 2d	Mock 5d	NaCl 5d
	**Conserved regulation**									
**Mode 1**	**A > D**	**A > D**	360 (8.14%)	762 (17.24%)	452 (10.23%)	702 (15.88%)	351 (7.94%)	776 (17.56%)	341 (7.71%)	779 (17.62%)
**Mode 2**	**A < D**	**A < D**	357 (8.08%)	790 (17.87%)	420 (9.50%)	758 (17.15%)	341 (7.71%)	785 (17.76%)	352 (7.96%)	791 (17.90%)
**Mode 3**	**A = D**	**A = D**	2645 (59.84%)	1633 (36.95%)	2648 (59.91%)	1679 (37.99%)	2648 (59.91%)	1552 (35.11%)	2640 (59.73%)	1524 (34.48%)
	**Subtotal**		3362 (76.06%)	3185 (72.06%)	3520 (79.64%)	3139 (71.02%)	3340 (75.57%)	3113 (70.43%)	3333 (75.41%)	3094 (70.00%)
	**Convergent regulation**									
**Mode 4**	**A > D**	**A = D**	425 (9.62%)	401 (9.07%)	305 (6.90%)	436 (9.86%)	435 (9.84%)	381 (8.62%)	413 (9.34%)	358 (8.10%)
**Mode 5**	**A < D**	**A = D**	279 (6.31%)	353 (7.99%)	232 (5.25%)	403 (9.12%)	297 (6.72%)	355 (8.03%)	298 (6.74%)	362 (8.19%)
	**Subtotal**		704 (15.93%)	754 (17.06%)	537 (12.15%)	839 (18.98%)	732 (16.56%)	736 (16.65%)	711 (16.09%)	720 (16.29%)
	**Divergent regulation**									
**Mode 6**	**A = D**	**A > D**	136 (3.08%)	210 (4.75%)	162 (3.67%)	210 (4.75%)	142 (3.21%)	235 (5.32%)	146 (3.30%)	289 (6.54%)
**Mode 7**	**A = D**	**A < D**	204 (4.62%)	260 (5.88%)	197 (4.46%)	220 (4.98%)	195 (4.41%)	316 (7.15%)	221 (5.00%)	296 (6.70%)
	**Subtotal**		340 (7.69%)	470 (10.63%)	359 (8.12%)	430 (9.73%)	337 (7.62%)	551 (12.47%)	367 (8.30%)	585 (13.24%)
	**Reversed regulation**									
**Mode 8**	**A > D**	**A < D**	11 (0.25%)	6 (0.14%)	3 (0.07%)	7 (0.16%)	10 (0.23%)	12 (0.27%)	6 (0.14%)	8 (0.18%)
**Mode 9**	**A < D**	**A > D**	3 (0.07%)	5 (0.11%)	1 (0.02%)	5 (0.11%)	1 (0.02%)	8 (0.18%)	3 (0.07%)	13 (0.29%)
	**Subtotal**		14 (0.32%)	11 (0.25%)	4 (0.09%)	12 (0.27%)	11 (0.25%)	20 (0.45%)	9 (0.20%)	21 (0.48%)

## Data Availability

All the transcriptomic data used in this study have been deposited previously at the National Center of Biotechnology Information under the accession PRJNA815810.

## Supplementary Materials

The following supporting information can be downloaded at: https://www.mdpi.com/article/10.3390/plants12173111/s1, Figure S1: Summary of DEGs on each chromosome; Table S1: Summary of the numbers of DEG between genotypes; Table S2: Summary of the proportions of DEGs between genotypes; Table S3: Summary of the number of DEGs and four homoeologous expression modes in EUP vs Mix; Table S4: Summary of the number of DEGs and homoeologous expression modes in X190 vs Mix; Supplementary Dataset.

## References

[1] Jiao Y., Wickett N.J., Ayyampalayam S., Chanderbali A.S., Landherr L., Ralph P.E., Tomsho L.P., Hu Y., Liang H., Soltis P.S. (2011). Ancestral polyploidy in seed plants and angiosperms. Nature.

[2] Fawcett J.A., Maere S., Van de Peer Y. (2009). Plants with double genomes might have had a better chance to survive the Cretaceous-Tertiary extinction event. Proc. Natl. Acad. Sci. USA.

[3] Soltis P.S., Soltis D.E. (2009). The role of hybridization in plant speciation. Annu. Rev. Plant Biol..

[4] Leitch A., Leitch I. (2008). Genomic plasticity and the diversity of polyploid plants. Science.

[5] McClintock B. (1984). The significance of responses of the genome to challenge. Science.

[6] Comai L., Madlung A., Josefsson C., Tyagi A. (2003). Do the different parental ‘heteromes’ cause genomic shock in newly formed allopolyploids?. Philos. Trans. R. Soc. B Biol. Sci..

[7] Chen Z.J., Ni Z. (2006). Mechanisms of genomic rearrangements and gene expression changes in plant polyploids. Bioessays.

[8] Doyle J.J., Flagel L.E., Paterson A.H., Rapp R.A., Soltis D.E., Soltis P.S., Wendel J.F. (2008). Evolutionary genetics of genome merger and doubling in plants. Annu. Rev. Genet..

[9] Buggs R.J., Zhang L., Miles N., Tate J.A., Gao L., Wei W., Schnable P.S., Barbazuk W.B., Soltis P.S., Soltis D.E. (2011). Transcriptomic shock generates evolutionary novelty in a newly formed, natural allopolyploid plant. Curr. Biol..

[10] Yoo M.-J., Liu X., Pires J.C., Soltis P.S., Soltis D.E. (2014). Nonadditive gene expression in polyploids. Annu. Rev. Genet..

[11] Edger P.P., Smith R., McKain M.R., Cooley A.M., Vallejo-Marin M., Yuan Y., Bewick A.J., Ji L., Platts A.E., Bowman M.J. (2017). Subgenome dominance in an interspecific hybrid, synthetic allopolyploid, and a 140-year-old naturally established neo-allopolyploid monkeyflower. Plant Cell.

[12] Bird K.A., VanBuren R., Puzey J.R., Edger P.P. (2018). The causes and consequences of subgenome dominance in hybrids and recent polyploids. New Phytol..

[13] Jiang X., Song Q., Ye W., Chen Z.J. (2021). Concerted genomic and epigenomic changes accompany stabilization of *Arabidopsis* allopolyploids. Nat. Ecol. Evol..

[14] Jackson S., Chen Z.J. (2010). Genomic and expression plasticity of polyploidy. Curr. Opin. Plant Biol..

[15] Rapp R.A., Udall J.A., Wendel J.F. (2009). Genomic expression dominance in allopolyploids. BMC Biol..

[16] Wang J., Tian L., Lee H.S., Wei N.E., Jiang H., Watson B., Madlung A., Osborn T.C., Doerge R.W., Comai L. (2006). Genomewide nonadditive gene regulation in Arabidopsis allotetraploids. Genetics.

[17] Yoo M.J., Szadkowski E., Wendel J.F. (2013). Homoeolog expression bias and expression level dominance in allopolyploid cotton. Heredity.

[18] Wang X., Zhang H., Li Y., Zhang Z., Li L., Liu B. (2016). Transcriptome asymmetry in synthetic and natural allotetraploid wheats, revealed by RNA-sequencing. New Phytol..

[19] Zhao N., Dong Q., Nadon B.D., Ding X., Wang X., Dong Y., Liu B., Jackson S.A., Xu C. (2020). Evolution of Homeologous Gene Expression in Polyploid Wheat. Genes.

[20] Liu Z., Adams K.L. (2007). Expression partitioning between genes duplicated by polyploidy under abiotic stress and during organ development. Curr. Biol..

[21] Dong S., Adams K.L. (2011). Differential contributions to the transcriptome of duplicated genes in response to abiotic stresses in natural and synthetic polyploids. New Phytol..

[22] Bardil A., de Almeida J.D., Combes M.C., Lashermes P., Bertrand B. (2011). Genomic expression dominance in the natural allopolyploid Coffea arabica is massively affected by growth temperature. New Phytol..

[23] Combes M.C., Cenci A., Baraille H., Bertrand B., Lashermes P. (2012). Homeologous gene expression in response to growing temperature in a recent Allopolyploid (*Coffea arabica* L.). J. Hered..

[24] Combes M.C., Dereeper A., Severac D., Bertrand B., Lashermes P. (2013). Contribution of subgenomes to the transcriptome and their intertwined regulation in the allopolyploid Coffea arabica grown at contrasted temperatures. New Phytol..

[25] Dong Y., Hu G., Grover C.E., Miller E.R., Zhu S., Wendel J.F. (2022). Parental legacy versus regulatory innovation in salt stress responsiveness of allopolyploid cotton (Gossypium) species. Plant J..

[26] Consortium I.W.G.S., Appels R., Eversole K., Stein N., Feuillet C., Keller B., Rogers J., Pozniak C.J., Choulet F., Distelfeld A. (2018). Shifting the limits in wheat research and breeding using a fully annotated reference genome. Science.

[27] Levy A.A., Feldman M. (2022). Evolution and origin of bread wheat. Plant Cell.

[28] Xiao J., Liu B., Yao Y., Guo Z., Jia H., Kong L., Zhang A., Ma W., Ni Z., Xu S. (2022). Wheat genomic study for genetic improvement of traits in China. Sci. China Life Sci..

[29] Sha Y., Li Y., Zhang D., Lv R., Wang H., Wang R., Ji H., Li S., Gong L., Li N. (2023). Genome shock in a synthetic allotetraploid wheat invokes subgenome-partitioned gene regulation, meiotic instability, and karyotype variation. J. Exp. Bot..

[30] Wang B., Lv R., Zhang Z., Yang C., Xun H., Liu B., Gong L. (2022). Homoeologous exchange enables rapid evolution of tolerance to salinity and hyper-osmotic stresses in a synthetic allotetraploid wheat. J. Exp. Bot..

[31] Hu G., Wendel J.F. (2019). Cis–trans controls and regulatory novelty accompanying allopolyploidization. New Phytol..

[32] Alger E.I., Edger P.P. (2020). One subgenome to rule them all: Underlying mechanisms of subgenome dominance. Curr. Opin. Plant Biol..

[33] Van de Peer Y., Mizrachi E., Marchal K. (2017). The evolutionary significance of polyploidy. Nat. Rev. Genet..

[34] Gou X., Bian Y., Zhang A., Zhang H., Wang B., Lv R., Li J., Zhu B., Gong L., Liu B. (2018). Transgenerationally precipitated meiotic chromosome instability fuels rapid karyotypic evolution and phenotypic diversity in an artificially constructed allotetraploid wheat (AADD). Mol. Biol. Evol..

[35] Lv R., Wang C., Wang R., Wang X., Zhao J., Wang B., Aslam T., Han F., Liu B. (2022). Chromosomal instability and phenotypic variation in a specific lineage derived from a synthetic allotetraploid wheat. Front. Plant Sci..

[36] Zhao J., Li J., Lv R., Wang B., Zhang Z., Yu T., Liu S., Xun H., Xu C., Wendel J.F. (2023). Meiotic pairing irregularity and homoeologous chromosome compensation cause rapid karyotype variation in synthetic allotetraploid wheat. New Phytol..

[37] Boideau F., Richard G., Coriton O., Huteau V., Belser C., Deniot G., Eber F., Falentin C., Ferreira de Carvalho J., Gilet M. (2022). Epigenomic and structural events preclude recombination in Brassica napus. New Phytol..

[38] Rönspies M., Schmidt C., Schindele P., Lieberman-Lazarovich M., Houben A., Puchta H. (2022). Massive crossover suppression by CRISPR–Cas-mediated plant chromosome engineering. Nat. Plants.

[39] Maherali H., Walden A.E., Husband B.C. (2009). Genome duplication and the evolution of physiological responses to water stress. New Phytol..

[40] Dong Y., Hu G., Yu J., Thu S.W., Grover C.E., Zhu S., Wendel J.F. (2020). Salt-tolerance diversity in diploid and polyploid cotton (Gossypium) species. Plant J..

[41] Xu S., Guo Z., Feng X., Shao S., Yang Y., Li J., Zhong C., He Z., Shi S. (2023). Where whole-genome duplication is most beneficial: Adaptation of mangroves to a wide salinity range between land and sea. Mol. Ecol..

[42] Lloyd A., Blary A., Charif D., Charpentier C., Tran J., Balzergue S., Delannoy E., Rigaill G., Jenczewski E. (2018). Homoeologous exchanges cause extensive dosage-dependent gene expression changes in an allopolyploid crop. New Phytol..

[43] Zhang Z., Xun H., Lv R., Gou X., Ma X., Li J., Zhao J., Li N., Gong L., Liu B. (2022). Effects of homoeologous exchange on gene expression and alternative splicing in a newly formed allotetraploid wheat. Plant J..

[44] Powell J.J., Fitzgerald T.L., Stiller J., Berkman P.J., Gardiner D.M., Manners J.M., Henry R.J., Kazan K. (2017). The defence-associated transcriptome of hexaploid wheat displays homoeolog expression and induction bias. Plant Biotechnol. J..

[45] Lee J.S., Adams K.L. (2020). Global insights into duplicated gene expression and alternative splicing in polyploid Brassica napus under heat, cold, and drought stress. Plant Genome.

[46] Van de Peer Y., Ashman T.-L., Soltis P.S., Soltis D.E. (2021). Polyploidy: An evolutionary and ecological force in stressful times. Plant Cell.

[47] Li M., Sun W., Wang F., Wu X., Wang J. (2021). Asymmetric epigenetic modification and homoeolog expression bias in the establishment and evolution of allopolyploid Brassica napus. New Phytol..

[48] Zhang Q., Guan P., Zhao L., Ma M., Xie L., Li Y., Zheng R., Ouyang W., Wang S., Li H. (2021). Asymmetric epigenome maps of subgenomes reveal imbalanced transcription and distinct evolutionary trends in Brassica napus. Mol. Plant.

[49] Rayburn A.L., Gill B. (1986). Molecular identification of the D-genome chromosomes of wheat. J. Hered..

[50] Kim D., Langmead B., Salzberg S.L. (2015). HISAT: A fast spliced aligner with low memory requirements. Nat. Methods.

[51] Love M.I., Huber W., Anders S. (2014). Moderated estimation of fold change and dispersion for RNA-seq data with DESeq2. Genome Biol..

[52] Benjamini Y., Hochberg Y. (1995). Controlling the false discovery rate: A practical and powerful approach to multiple testing. J. R. Stat. Soc. Ser. B (Methodol.).

[53] Emms D.M., Kelly S. (2019). OrthoFinder: Phylogenetic orthology inference for comparative genomics. Genome Biol..

[54] Wang Y., Tang H., DeBarry J.D., Tan X., Li J., Wang X., Lee T.-h., Jin H., Marler B., Guo H. (2012). MCScanX: A toolkit for detection and evolutionary analysis of gene synteny and collinearity. Nucleic Acids Res..

