# Supplementary material for: Effects of Allopolyploidization and Homoeologous Chromosomal Segment Exchange on Homoeolog Expression in a Synthetic Allotetraploid Wheat under Variable Environmental Conditions
Source: Plants (Basel). 2023 Aug 30;12(17):3111. doi: 10.3390/plants12173111 (PMC10490264; doi:10.3390/plants12173111)
Supplement: Supplementary file 1 [file plants-12-03111-s001.zip › Supplementary Tables.pdf]

Table S1. Summary of the numbers of DEG between genotypes.

Comparison	Condition	A-DEG	D-DEG	Up-regulated	Down-regulated	Total
EUP vs Mix	Mock-2day	1807	3410	2331	2886	5217
	PEG6000-2day	2321	3787	2840	3268	6108
	Mock-5day	567	760	691	636	1327
	NaCl-5day	1120	1845	1393	1572	2965
X190 vs Mix	Mock-2day	2361	3620	2324	3657	5981
	PEG6000-2day	1979	4005	3229	2755	5984
	Mock-5day	3184	4694	3953	3925	7878
	NaCl-5day	1748	4624	3576	2796	6372

Table S2. Summary of the proportions of DEG between genotypes.

Comparison	Condition	A-DEG	D-DEG	Up-regulated	Down-regulated	Total
EUP vs Mix	Mock-2day	0.120	0.185	0.136	0.169	0.305
	PEG6000-2day	0.155	0.206	0.168	0.192	0.360
	Mock-5day	0.038	0.041	0.041	0.038	0.079
	NaCl -5day	0.075	0.100	0.083	0.092	0.175
X190 vs Mix	Mock-2day	0.157	0.196	0.138	0.216	0.354
	PEG6000-2day	0.132	0.217	0.189	0.160	0.349
	Mock-5day	0.212	0.255	0.234	0.233	0.467
	NaCl -5day	0.116	0.251	0.207	0.160	0.367

Table S3. Summary of the number of DEGs and four homoeolgous expression modes in EUP vs Mix

	EUP vs Mix															
	Mock（2d）				PEG6000（2d）				Mock（5d）				NaCl（5d）			
Type	Mode 4	Mode 5	Mode 6	Mode 7	Mode 4	Mode 5	Mode 6	Mode 7	Mode 4	Mode 5	Mode 6	Mode 7	Mode 4	Mode 5	Mode 6	Mode 7
A↑ D↑	10	2	0	2	8	1	6	1	0	0	0	0	1	5	0	0
A= D↑	128	0	0	33	100	0	0	45	14	0	0	5	41	0	0	14
A↓ D↑	5	0	0	2	0	0	0	2	0	0	0	0	5	0	0	2
A= D↓	0	51	24	0	0	78	47	0	0	13	11	0	0	66	35	0
A↓ D↓	3	2	4	3	7	6	5	4	0	0	0	0	2	1	2	0
A↓ D=	64	0	0	39	81	0	0	62	25	0	0	15	82	0	0	46
A↑ D↓	0	0	1	0	0	1	1	0	0	0	0	0	0	1	0	0
A↑ D=	0	17	10	0	0	69	31	0	0	27	9	0	0	56	13	0
A= D=	215	207	97	125	205	198	120	146	266	192	142	177	305	274	160	158

Table S4. Summary of the number of DEGs and four homoeologous expression modes in X190 vs Mix

	X190 vs Mix															
	Mock（2d）				PEG6000（2d）				Mock（5d）				NaCl（5d）			
Type	Mode 4	Mode 5	Mode 6	Mode 7	Mode 4	Mode 5	Mode 6	Mode 7	Mode 4	Mode 5	Mode 6	Mode 7	Mode 4	Mode 5	Mode 6	Mode 7
A↑ D↑	3	0	2	3	9	1	3	5	26	17	7	8	5	1	1	3
A= D↑	77	0	0	21	111	0	0	56	77	0	0	46	133	0	0	99
A↓ D↑	4	0	0	0	5	0	0	3	8	0	0	2	3	0	0	3
A= D↓	0	49	22	0	0	81	44	0	0	35	34	0	0	127	108	0
A↓ D↓	12	6	6	8	3	9	1	2	21	8	6	11	0	3	3	2
A↓ D=	89	0	0	53	61	0	0	59	99	0	0	59	32	0	0	33
A↑ D↓	0	1	0	0	0	3	4	0	0	1	0	0	0	3	1	0
A↑ D=	0	35	12	0	0	67	37	0	0	78	32	0	0	38	26	0
A= D=	250	206	100	110	192	194	146	191	182	159	67	95	185	190	150	156

A↑, the A homeolog was upregulated, A↓, the A homeolog was downregulated, A=, the A homeolog was unchanged, D↑, the D homeolog was upregulated, D↓, the D homeolog was downregulated, D=, the D homeolog was unchanged.
