# Supplementary figures and images for: Effects of Allopolyploidization and Homoeologous Chromosomal Segment Exchange on Homoeolog Expression in a Synthetic Allotetraploid Wheat under Variable Environmental Conditions
Source: Plants (Basel). 2023 Aug 30;12(17):3111. doi: 10.3390/plants12173111 (PMC10490264; doi:10.3390/plants12173111)

**Figure S1. Summary of DEGs on each chromosome.**

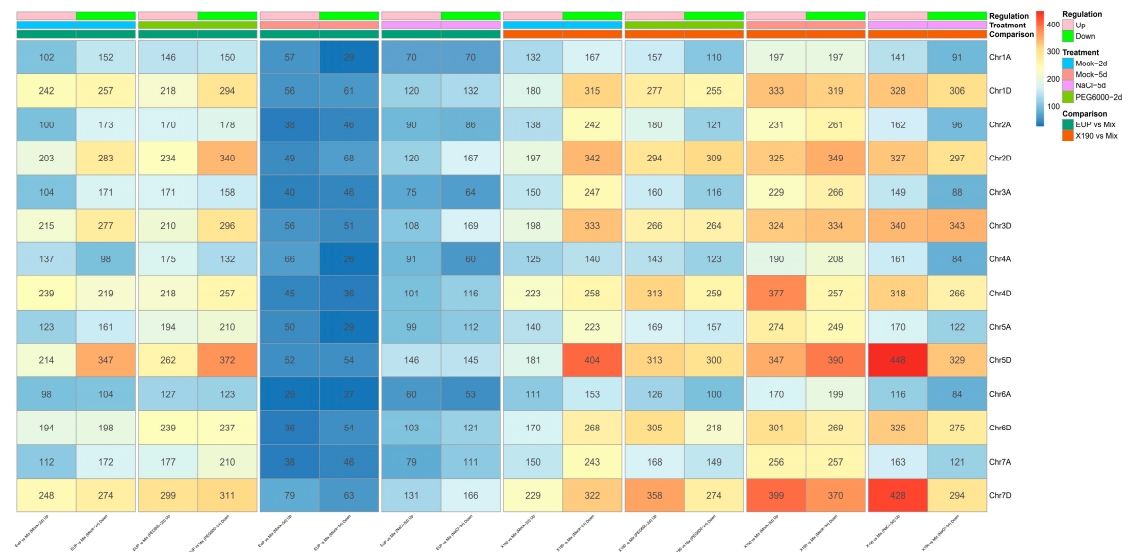

Supplement: Supplementary file 1 [file plants-12-03111-s001.zip › Supplementary Figures.pdf]
